# The Association Between COVID-19 and Thyroxine Levels: A Meta-Analysis

**DOI:** 10.3389/fendo.2021.779692

**Published:** 2022-01-04

**Authors:** Yiru Chen, Xiuneng Li, Yu Dai, Jingjing Zhang

**Affiliations:** National Clinical Research Center for Metabolic Diseases, Metabolic Syndrome Research Center, Key Laboratory of Diabetes Immunology, Ministry of Education, and Department of Metabolism and Endocrinology, The Second Xiangya Hospital of Central South University, Changsha, China

**Keywords:** FT3, FT4, TSH, COVID-19, mortality

## Abstract

**Objectives:**

Recently, a number of reports have described the potential relationship between COVID-19 and thyroid hormones, but the results were conflicting. We performed a meta-analysis to evaluate the effect of the severity of COVID-19 on thyroid-related hormones and the effect of thyroid-related hormones on the outcome of COVID-19 in order to try to confirm the association between the serum levels of free triiodothyronine (FT3), free thyroxine (FT4) and thyroid stimulating hormone (TSH) and the severity or mortality of coronavirus-19 patients.

**Methods:**

The methodology was already registered in the International Prospective Register of Systematic Reviews (PROSPERO) database, and the protocol number is CRD42021269246. Systematic searches were carried out on the Cochrane Library, Embase, PubMed and Web of Science databases on November 15, 2021. We set up the literature search strategy based on the following keywords: [(T3 OR FT3 OR triiodothyronine) or (T4 OR FT4 OR thyroxine) or (TSH or thyrotropin)] and (COVID-19 OR SARS-CoV-2), without time restrictions.

**Results:**

Twenty studies satisfied the inclusion/exclusion criteria and were included in the meta-analysis. A total of 3609 patients were enrolled in the study. From the analysis of the included studies, the incidence of thyroid-related hormone abnormalities was higher in patients with severe COVID-19, and the serum levels of FT3 and TSH were lower than those of patients with nonsevere COVID-19. However, the difference in the FT4 levels was not significant. Similar characteristics were shown between survivors and nonsurvivors. In addition, the outcomes of the meta-analysis showed that patients with abnormal thyroid-related hormones had greater mortality.

**Conclusions:**

Low FT3 serum levels, low FT4 serum levels and low TSH serum levels may increase the mortality of COVID-19 patients during admission. On the other hand, the higher the severity level of COVID-19, the higher the probability of decreases in the FT3, FT4, TSH levels.

## 1 Introduction

The outbreak of COVID-19 pneumonia has had a great impact on the global community, and it has challenged the capacity of health care systems in all countries. Existing studies have revealed that SARS-COV-2 could influence the glucose and lipid levels and the blood pressure through metabolic and endocrine pathways (1–3) in which angiotensin-converting enzyme 2 (ACE2) plays a key role. ACE2 was originally found to be a functional receptor for the SARS coronavirus in 2003 (4), and it is highly expressed in the thyroid gland in humans (5), which is one of the potential mechanisms by which COVID-19 leads to thyroid dysfunction.

The hormones involved in the hypothalamus-pituitary-thyroid axis include thyrotropin-releasing hormone (TRH), thyroid stimulating hormone (TSH), free triiodothyronine (FT3) and free thyroxine (FT4). Currently, the relationship between thyroid gland function and COVID-19 remains unclear. In 2020, in Runmei Zou’s study, it was found that 27.52% of patients with COVID-19 had thyroid disease (6). Moreover, in a study based on severe cases in the UK, the proportion of thyroid follicular epithelial cells that were damaged was 22.2% (7). It is currently believed that COVID-19 has a direct effect on thyroid function and the thyroid hormone levels through the hypothalamus-pituitary-thyroid axis and can also affect the thyroid gland by autoimmune diseases through cytokines (8). It has been found that the TSH levels are negatively correlated with the mortality of COVID-19 in patients with normal FT3 and FT4 levels (9). The TSH and FT4 levels were low in confirmed COVID-19-positive patients during admission, and they returned to normal levels when the patient recovered (10). The meta-analysis of M. Llamas has shown that the level of FT3 is closely related to the severity of COVID-19 (11). The correlation between the thyroid hormone level and mortality, severity and prognosis of patients with COVID-19 still need to be systematically described. This meta-analysis focuses on these problems and aims to guide the clinical classification and treatment of patients with COVID-19, and the effect of the thyroid hormone levels in COVID-19 patients with previously normal thyroid function was assessed.

## 2 Materials and Methods

### 2.1 Protocols and Registration

Our material and methods were based on the Preferred Reporting Items for Systematic Reviews and Meta-Analyses (PRISMA) guidelines (12), which have already been registered in the International Prospective Register of Systematic Reviews (PROSPERO) database, and the protocol number is CRD42021269246.

### 2.2 Eligibility Criteria

All original peer-reviewed research publications were taken into consideration. We developed the following criteria to select eligible studies: (I) population:patients with COVID-19; (II) intervention: no; (III) comparator/control: ICU patients and non ICU patients were compared with each other; (IV) outcomes: studies in which the serum levels FT3, FT4 or TSH of the different groups (severe *vs*. nonsevere or survivor *vs*. nonsurvivor) in the form of mean ± SD or median with interquartile range (IQR) were available, the in-hospital mortality data of COVID-19 patients with low FT3 or FT4 or TSH serum levels were available, or the occurrence rate data of low FT3, FT4 and TSH levels in severe and patients with nonsevere COVID-19 were available or in survivors and nonsurvivors could be obtained; (V) study design: clinical studies.

Patients in the study who were hospitalized with a confirmed COVID-19 diagnosis were defined as having certain COVID-19 severities in accordance with the Clinical Guidelines for the Diagnosis and Treatment of COVID-19 in China (6th Edition). Studies involving patients with a history of thyroid disease or patients receiving or receiving treatment with a potential impact on thyroid function were excluded. An NTIS state was defined as patients with FT3<3.3 pmol/L, whose FT4 level was low or normal and with TSH levels of 0.35-4.8 mIU/L.

### 2.3 Information Sources and Search Strategy

We systematically searched the Cochrane Library, Embase, PubMed and Web of Science databases in November 2021 without time restrictions during the search for all published articles related to both thyroid-related hormones and COVID-19. The literature search strategy was based on the following keywords: [(T3 OR FT3 OR triiodothyronine) or (T4 OR FT4 OR thyroxine) or (TSH or thyrotropin)] and (COVID-19 OR SARS-CoV-2 OR 2019 novel coronavirus). In addition, to identify articles other than those found in the electronic databases, a further manual search of studies meeting our inclusion criteria was also performed. Two independent reviewers (YC and XL) performed the first step of the title/abstract screening and the second step of full-text assessment in the search process, and any disagreement that arose during this process was discussed until an agreement was reached.

### 2.4 Study Selection

After obtaining the list of all relevant articles, we removed duplicate articles and nonclinical research and also excluded a series of studies with poor correlations. Two reviewers (YC and XL) independently selected eligible studies for inclusion by reading the titles and abstracts. Disagreements were resolved by reaching a consensus or with the help of a third reviewer (YD).

### 2.5 Data Extraction

According to the inclusion/exclusion criteria, the full texts of all potentially qualified studies were independently reviewed by two reviewers (YC) and (XL). Disagreements were addressed through discussion. If a consensus could not be reached, a third reviewer (YD) resolved the disagreements. Information including the author, country, type of study, sample size, mean or median age, sex ratio, population and NOS scores was extracted from the selected studies. All of the extracted data were tabulated.

### 2.6 Quality Assessment of Studies

All the articles included in this meta-analysis were evaluated by the Newcastle Ottawa Scale (NOS) (13). We scored all 20 studies from the perspectives of the study type, inclusion criteria of COVID-19 patients, sample size, follow-up time, index detection, and comparability between the experimental group and the control group. Articles with a score of 6 or more were defined as high-quality articles. All of the authors reached a consensus on the disagreement on the quality of the studies through discussion and consultation.

### 2.7 Statistical Analysis

All data of this meta-analysis were analyzed in RevMan (version 5.4) and Stata (version 15.1). For all included studies, the risk ratio (RR) and 95% confidence interval (Cl) were used to measure the correlation between the TSH, FT3, and FT4 levels and the mortality and severity of COVID-19 patients. The I^2^ value and P value were used to evaluate heterogeneity. If the I^2^ was less than 50% and if the P value was less than 0.05, there was no heterogeneity in the included studies. In addition, sensitivity analyses were used to ensure that the method we adopted was scientific (**Supplementary Figures 1**–**10**).

## 3 Results

### 3.1 Search Results and Studies Characteristics

**Figure 1:**
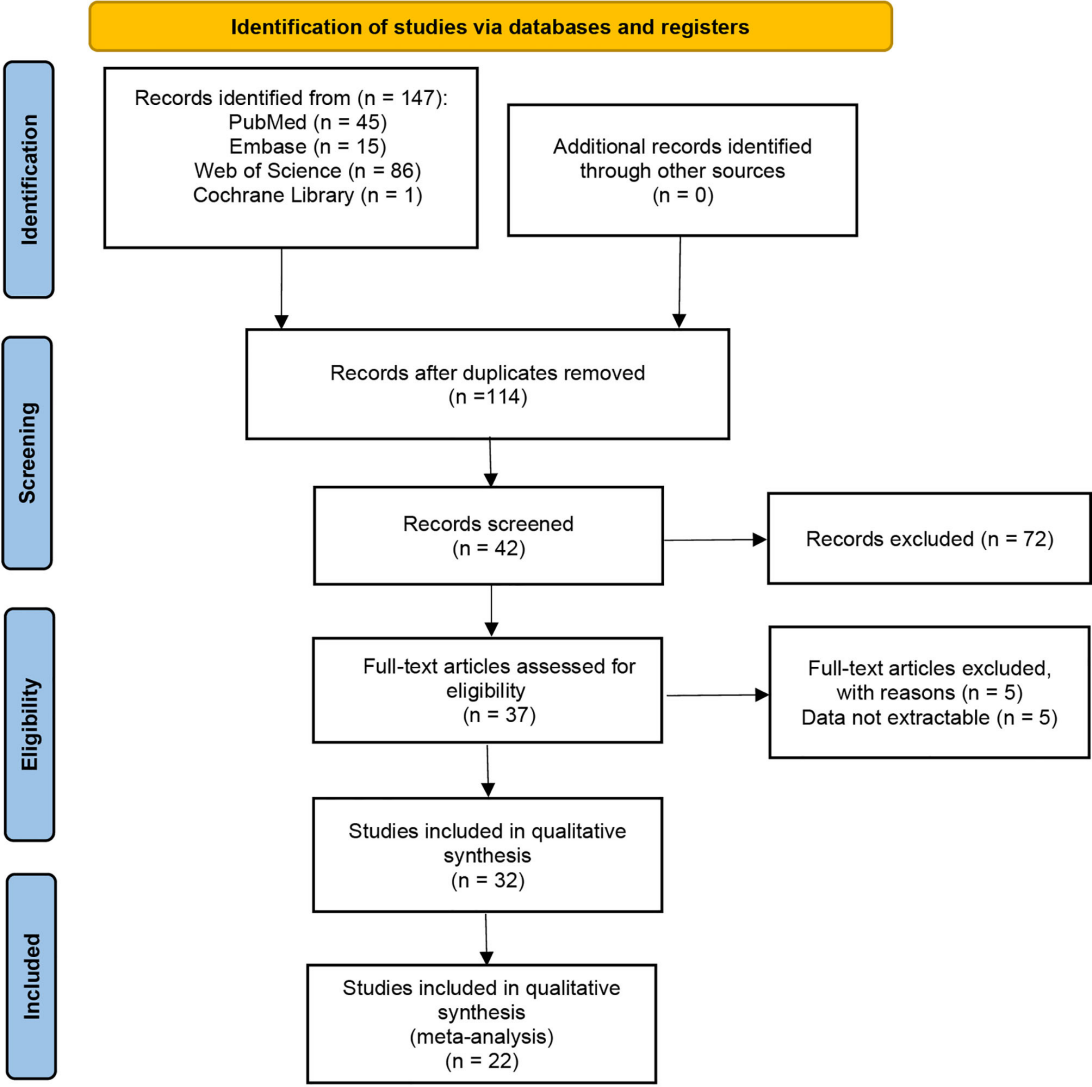
Flow diagram of the study selection process.

147 studies were identified in the literature search, and twenty retrospective studies satisfying our inclusion/exclusion criteria were involved in the meta-analysis, as shown in **Figure 1**. The involved studies had a total of 3609 patients. Sufficient details of the involved studies are shown in **Table 1**.

**Table 1:** Description of eligible studies reporting the association between thyroid-related hormones and COVID-19.

NO.	Author	Country	Type of Study	Sample Size	Age	Male	Population
1	Andrea Lania (14)	Italy	retrospective study	287	66 (27–92)	193	patients with COVID-19
2	Baldelli et al. (15)	Italy	retrospective study	66	60.8 ± 17.0 58.4 ± 12.1	34	with COVID-19 pneumonia/ICU patients
3	Campi et al. (16)	Italy	retrospective study	144	68.1 ± 14.67	97	patients with COVID-19
4	Chen et al. (17)	China	retrospective study	50	48.4 ± 13.7	33	patients with COVID-19
5	Gao et al. (18)	China	retrospective study	100	61.4 ± 15.2 63.2 ± 13.4	52	patients with non-severe COVID-19 severe or critically ill patients with COVID-19
6	Gong et al. (9)	China	retrospective study	150	69.5 (IQR:61-79)	81	patients with COVID-19
7	Güven and Gültekin (19)	Turkey	prospective study	250	68 (IQR:54- 78)	157	patients with COVID-19 in non-ICU and patients with COVID-19 in ICU.
8	Khoo et al. (10)	UK	cohort observational study	456	66.1± 16.0 63.8± 19.3	271	patients with COVID-19 and patients without COVID-19
9	Lui et al. (20)	Hong Kong, China	cohort study	191	53.5 ± 17.2	99	patients with COVID-19
10	Lui et al. (21)	Hong Kong, China	prospective study	367	54(IQR:38-65)	172	patients with COVID-19
11	Schwarz et al. (22)	Israel	retrospective study	54	Unknown	37	patients with COVID-19
12	Lang et al. (23)	China	retrospective study	127	66 (53–71)	62	patients with COVID-19
13	Vassiliadi et al. (24)	Greece	cohort observational study	196	59.3 ± 18.3	130	patients with COVID-19 and patients without COVID-19
14	Zou et al. (6)	China	retrospective study	149	47 (36, 61.5)	71	patients with COVID-19
15	Chen et al. (25)	China	retrospective study	274	62.0(44.0-70.0)	171	patients with COVID-19
16	Dutta et al. (26)	India	retrospective study	236	54(15-91)	159	patients with COVID-19
17	Beltrão et al. (27)	Brazil	retrospective study	245	62(49-74.5)	100	patients with COVID-19
18	Ahn et al. (28)	Korea	retrospective study	119	64.3 ± 16.8	62	patients with COVID-19
19	Ballesteros Vizoso et al. (29)	Spain	retrospective study	78	59 ± 12 68 ± 12	55	patients with COVID-19
20	Clarke et al. (30)	UK	prospective study	70	55.9 ± 13	47	patients with COVID-19

### 3.2 Quality Assessment

The quality of the included studies was assessed by the Newcastle-Ottawa Scale (NOS). The NOS scale is mainly composed of the sample selection, exposure and comparability between the experimental group and the control group in the studies. The scores of all the extracted studies scored with the NOS scale were higher than 6, indicating the high quality of the included studies. The risk of bias is described in **Table 2**.

**Table 2:** Quality scores of included studies using newcastle-ottawa scale.

NO.	First author	Year	Selection	Comparability	Outcome	NOS
1	Andrea Lania (14)	2020	**	*	***	6*
2	Baldelli, R (15)	2021	****	**	***	9*
3	Campi, I (16)	2021	***	**	**	7*
4	Chen, M (17)	2021	****	**	***	9*
5	Gao, W (18)	2021	****	**	***	9*
6	Gong, J (9)	2021	****	**	***	9*
7	Güven, M (19)	2021	***	*	***	7*
8	Khoo, B (10)	2021	***	**	***	8*
9	Lui, David Tak Wai (1) (20)	2021	****	**	***	9*
10	Lui, David Tak Wai (2) (21)	2021	***	**	***	8*
11	Schwarz,Y (22)	2021	****	**	***	9*
12	Shan Lang (23)	2021	****	**	***	9*
13	Vassiliadi, Dimitra (24)	2021	***	**	***	8*
14	Runmei Zou (6)	2020	****	**	***	9*
15	Tao Chen (25)	2020	***	**	***	8*
16	Aditya Dutta (26)	2021	****	**	***	9*
17	Beltrão FEL (27)	2021	****	**	***	9*
18	Jiyeon Ahn (28)	2021	***	**	***	8*
19	Ballesteros Vizoso MA (29)	2021	****	**	***	9*
20	Clarke SA (30)	2021	****	**	***	9*

Selection: * :Meet the one item in the NOS selection section. ** :Meet the two items in the NOS selection section. *** : Meet the three items in the NOS selection section. **** :Meet the four items in the NOS selection section.

Comparability: * :The comparability of the study cohort design was of medium quality. ** :The comparability of the study cohort design was of high quality.

Outcome: * :Meet the one item in the NOS outcome section. ** :Meet the two items in the NOS outcome section. *** : Meet the three items in the NOS outcome section.

### 3.3 Meta-Analysis

A total of 20 articles were included in this meta-analysis. The basic characteristics and the scores of these studies assessed with the NOS scale are described in **Table 1**. A total of 3609 COVID-19 hospitalized patients were included in our study, and the patients ages ranged from 15 to 92 years old (14). Among the 20 studies in this meta-analysis, eight studies were conducted in China, three in Italy, two in the United Kingdom and the other seven in Greece, Turkey, India, Brazil, Korea, Spain and Israel. Three of them were published in 2020 and 17 in 2021. Among the 20 studies, there were 2083 male patients, accounting for 57.72% of the included patients. All sensitivity analyses are available in the **Supplementary Materials** (**Supplementary Figures 1**–**10**).

### 3.4 Primary Outcomes

#### 3.4.1 Thyroid-Related Hormones Levels and Survival Status of COVID-19 Patients

**Figure 2:**
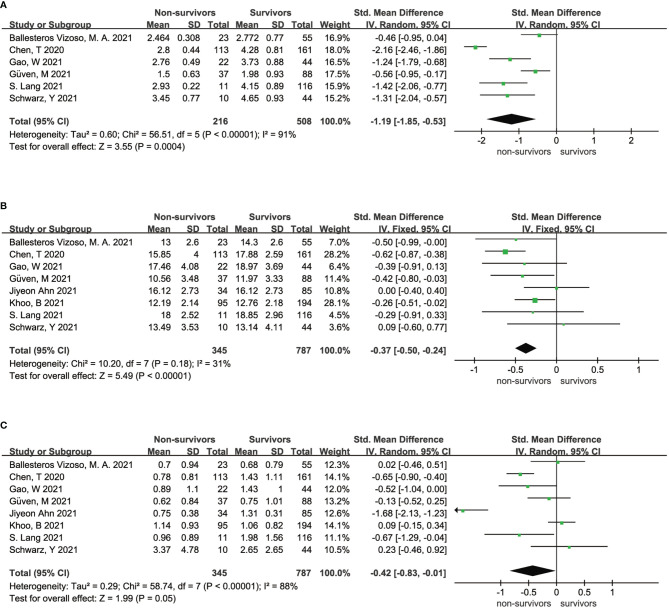
Forest plot comparing the FT3 serum levels **(A)**, FT4 serum levels **(B)**, and TSH serum levels **(C)** between the survivors and nonsurvivors.

High heterogeneity existed in both the analysis of the association between FT3 and the survival state of COVID-19 patients (I^2^ = 91%, P < 0.01) and the analysis of the association between TSH and the survival state of COVID-19 patients (I^2^ = 88%, P < 0.01) (**Figures 2A, C**), indicating that the included articles in these analyses failed to provide reliable information for further analysis. However, the overall data showed that survivors had a higher FT4 level than nonsurvivors (SMD = - 0.37 95% CI, -0.50 - -0.24), which is shown in **Figure 2B**. Sensitivity analyses were also performed to explore the potential sources of heterogeneity (**Supplementary Figures 1**–**3**).

#### 3.4.2 Thyroid-Related Hormones Levels and Severity of COVID-19

**Figure 3:**
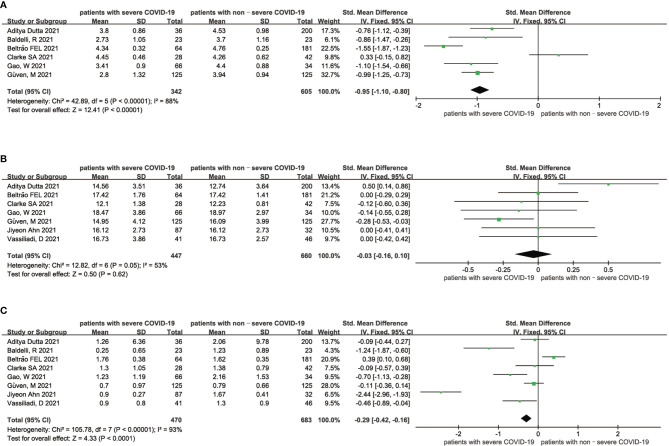
Forest plot comparing the FT3 serum levels **(A)**, FT4 serum levels **(B)**, and TSH serum levels **(C)** between the severe patients and nonsevere patients with COVID-19.

As shown in **Figure 3**, high heterogeneity existed in both the analysis of the association between FT3 and the severity of COVID-19 patients (I^2^ = 88%, P < 0.01) and the analysis of the association between TSH and the severity of COVID-19 patients (I^2^ = 93%, P < 0.01) (**Figures 3A, C**), indicating that the included articles in these analyses failed to provide reliable information for further analysis. The heterogeneity of the correlation analysis between FT4 and the severity of COVID-19 patients cannot also be ignored (I^2^ = 53%) (**Figure 3B**). Sensitivity analyses were also performed to explore the potential sources of heterogeneity (**Supplementary Figures 6**–**8**).

#### 3.4.3 Probability of Low Thyroid-Related Hormones Levels and Severity of COVID-19 Patients

**Figure 4:**
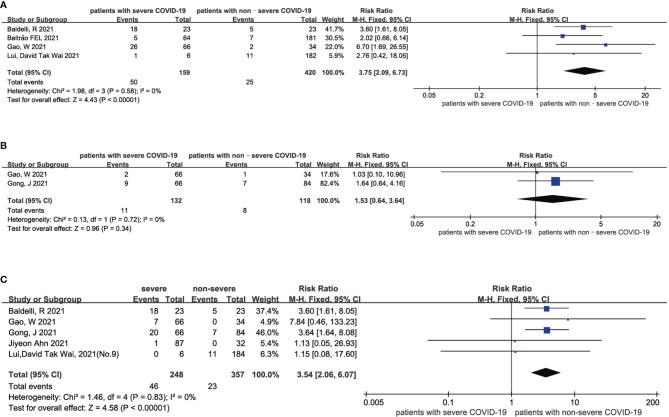
Forest plot comparing the probability of low FT3 **(A)**, low FT4 **(B)**, and low TSH **(C)** between the severe patients and nonsevere patients with COVID-19.

The results of this meta-analysis of the probability of low thyroid-related hormone levels and the severity of COVID-19 patients are shown in **Figure 4**. In general, the probability of low FT3 (RR = 3.75 95% CI, 2.09-6.73), FT4 (RR = 1.53 95% CI, 0.64-3.64) and TSH (RR = 3.54 95% CI, 2.06-6.07) levels was associated with a more severe COVID-19 disease. The statistical results showed that there was no significant heterogeneity among the studies. The sensitivity analysis also indicated the stability of our results (**Supplementary Figures 9**, **10**).

#### 3.4.4 Mortality in COVID-19 Patients With Non-Thyroidal Illness Syndrome (NTIS) and Without NTIS

**Figure 5:**
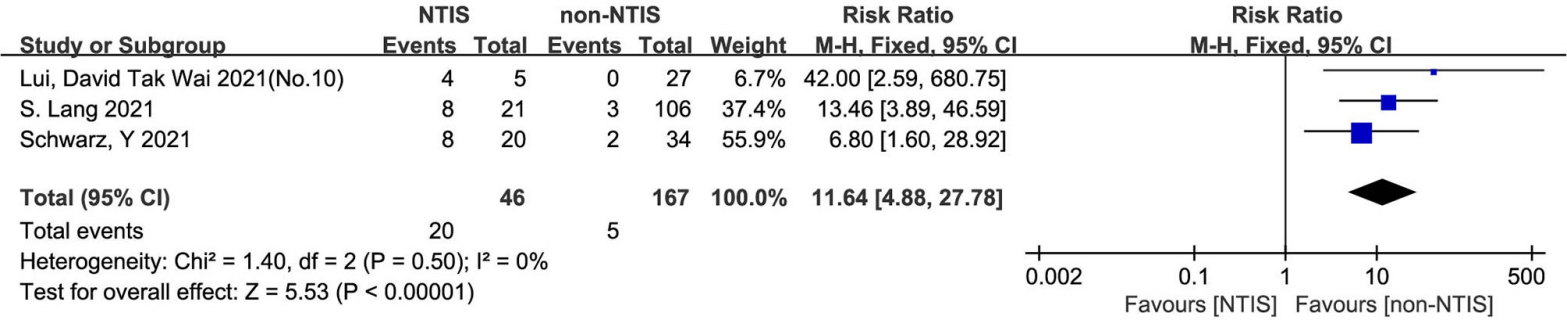
Forest plot for all studies comparing the mortality in the NTIS and non-NTIS patients with COVID-19.

The heterogeneity test results of the three studies included in this study were calculated as X^2^ = 1.40, df = 2, I^2^ = 0% and P = 0.30 in the Q-test, and the test showed no significant heterogeneity among the three records. The risk ratio of the three records was 11.64, 95% CI (4.88, 27.78), and the results were distinct (Z = 5.53, P < 0.01), revealing that the mortality of COVID patients with NTIS was higher than that of non-NTIS patients (**Figure 5**).

#### 3.4.5 Mortality in COVID-19 Patients With Low TSH and Normal TSH

**Figure 6:**
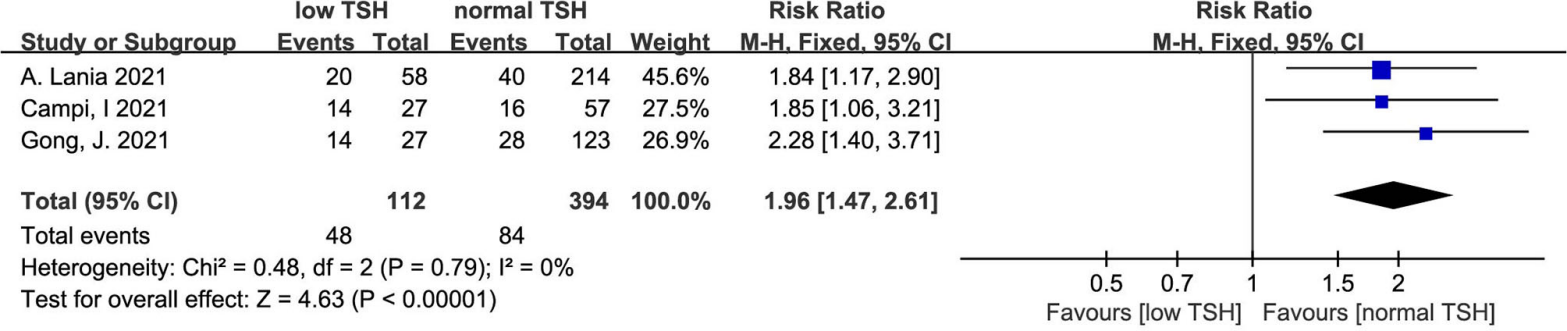
Forest plot for all studies comparing the mortality in the COVID-19 patients with low TSH levels and normal TSH levels.

The heterogeneity test results of the three studies included in this study were calculated as X^2^ = 0.48, df = 2, I^2^ = 0% and P =0.79 in the Q-test, and the results showed that there was no significant heterogeneity among the three records. The risk ratio of the three records was 1.96, 95% CI (1.47, 2.61), and the results were distinct (Z = 4.63, P < 0.01), revealing that the mortality of COVID patients with low TSH levels was higher than that of patients with normal TSH levels (**Figure 6**).

## 4 Discussion

There is little literature in the world on the relationship between thyroxine and the COVID-19 virus thus far. Existing studies have revealed complex interactions between the thyroid gland and viruses through hormones and signaling molecules (31). However, the effect of the COVID-19 virus on the level of thyroid hormone and the mechanisms involved remain unclear.

Our meta-analysis mainly indicates that low FT4 levels may be associated with adverse outcomes (**Figure 2B**) and severe COVID-19 (**Figure 3B**). Low FT3 serum levels may also increase the degree of severity of COVID-19 (**Figure 3A**). Correspondingly, we found that NTIS (**Figure 5**) or low TSH (**Figure 6**) serum levels might also increase the mortality of COVID-19 patients and that patients with severe COVID-19 had a higher probability of low thyroid-related hormone levels (**Figure 4**). The data above may be due to the “cytokine storm” induced by SARS-COV-2 infection, which leads to the development of autoimmune thyroiditis (32) and thus supports the role of FT3 and FT4 as prognostic biomarkers in COVID-19 patients. Also, the severity of SARS-COV-2 might be the dominant determinant of thyroid dysfunction (33).

The number of patients included in this study is limited, as few relevant studies in the world could be obtained, which leads to insufficient evidence currently supporting the interaction between thyroid-related hormones and the COVID-19 virus. Changes in the iodothyronine deiodinase levels, TSH secretion, the binding of thyroid hormone to plasma proteins, the transport of thyroid hormones in the peripheral tissues, and changes in the thyroid hormone receptor activity are all likely to contribute to the changes in serum levels of thyroid-related hormones in COVID-19 patients, but this needs further investigation. The severity of COVID-19 begins with the binding of the spike protein, which is on the surface of the virus, to the ACE2 receptor on the surface of the tissue cell (34). ACE2 is widely expressed in arteriovenous endothelial cells of many organs, especially in the thyroid gland (19). Studies have shown that the destruction of the thyroid gland (HPT axis) by SARS-COV-2 involves thyroid disease and the changes in related hormones (33). There are two possible mechanisms to explain the changes in the hypothalamic-pituitary-thyroid (HPT) axis in COVID-19 patients (33). First, the abnormal systemic inflammatory-immune responses caused by SARS-COV-2 (severe acute respiratory syndrome coronavirus 2) infection causes an indirect effect on the HPT axis. The presence of SARS-COV-2 RNA in the serum and plasma of COVID-19 patients, as well as the expression of ACE2 by the hypothalamus and pituitary gland, support this theory (5, 35). Second, the virus directly effects the thyroid gland. SARS-COV-2 attacks the lungs as well as other organs, including the thyroid gland (7, 36). Vojdani and coworkers also provide molecular evidence that SARS-COV-2 antibodies react with the thyroid gland (37). In addition, disruption of thyroid follicles and parafollicular cells was clearly observed in autopsies of patients who died of COVID-19 (38), which is a typical histopathological feature of thyroid injury. Molecular analysis of thyroid surgical specimens showed that thyroid follicular cells expressed ACE2 (39), suggesting that the thyroid gland is vulnerable to SARS-COV-2 damage once the patient is infected.

Abnormalities of thyroid function may represent an isolated change, but they can also be a precursor to autoimmune polyglandular syndrome or endocrine disorders (40). On the one hand, patients with severe COVID-19 do not have the typical characteristics of patients with subacute thyroiditis and instead have reduced white blood cell levels (41). However, SARS-COV-2-induced thyroid dysfunction is consistent with syndromes of NTIS. Our meta-analysis suggests that such COVID-19 patients require intensive care and are at risk for thyrotoxicosis (**Figure 4**). On the other hand, studies have suggested that hyperthyroidism or hypothyroidism might increase the risk of developing a severe course of COVID-19 (42), as SARS-COV2 is able to enter the host cell by ACE-2. Therefore, an abnormal thyroid function would increase the burden of cardiovascular (43, 44) and psychiatric (45) comorbidities. Although the World Health Organization (WHO) did not recommend systematic thyroid function tests for hospitalized COVID-19 patients in March 2020 (12), the proportion of patients with severe COVID-19 who have abnormal serum-associated thyroxine levels is higher than patients with severe COVID-19 who did not have abnormal serum thyroxine levels according to the results of our study, and it is necessary to conduct thyroid function tests for COVID-19 patients, especially those admitted to the emergency room or intensive care unit (ICU), to avoid worsening outcomes (42). For severe or critically ill patients, low FT3 and TSH levels could be regarded as a type of adaptation to NTIS caused by major stress conditions such as systemic viral diseases, which could include SARS (13).

## 5 Strength and Limitations

A total of 20 studies were included in this meta-analysis, and these were mainly retrospective studies. We searched the PubMed, EMBASE, Web of Science, and Cochrane Library databases. A total of 3069 patients were included, including patients with nonsevere COVID-19 and patients with severe COVID-19, and the study included survivors and nonsurvivors. We summarized the relationship between TSH, FT3, FT4 and COVID-19 through scientific sorting and meta-analysis. To our knowledge, this is the first study to comprehensively summarize the relationship between levels thyroid-related hormones and COVID-19 by using a meta-analysis.

Our meta-analysis has some limitations, which must be carefully considered when interpreting the results. There were some heterogeneities among the studies included in this meta-analysis, and the sample size of the included studies was relatively insufficient after reasonable sorting. The current research regarding the relationship between thyroid-related hormones and COVID-19 is limited. Some of the included studies did not exclude the influence of other related factors (such as renal disorders) when examining thyroid function.

## 6 Conclusions

In summary, our study revealed that low serum levels of thyroid-related hormones may increase the mortality of COVID-19 patients during admission, and the higher the severity of COVID-19 is, the higher the probability of a decrease in the FT3, FT4, and TSH levels. Our findings could provide clinical guidance for thyroid function detection in patients with severe COVID-19. Further randomized controlled trials are needed to confirm these findings.

## Data Availability Statement

The original contributions presented in the study are included in the article/**Supplementary Material**. Further inquiries can be directed to the corresponding author.

## Author Contributions

Conceptualization: YC. Methodology: YC and XL. Software: YC and XL. Validation: YC and XL. Formal analysis: YC and XL. Investigation: YC and XL. Data curation: YC, XL, and YD. Writing—review and editing: YC, XL, and YD. Visualization: JZ. Supervision: YD and JZ. Funding acquisition: JZ. All authors contributed to the article and approved the submitted version.

## Funding

This work was supported by grants from the National Natural Science Foundation of China (82070807, 91749118, 81770775, 81730022), Natural Science Foundation of Hunan Province, China (2021JJ30976) and National key research and development program (2019YFA0801903, 2018YFC2000100) and College Student Innovation and Entrepreneurship Training Program.

## Conflict of Interest

The authors declare that the research was conducted in the absence of any commercial or financial relationships that could be construed as a potential conflict of interest.

## Publisher’s Note

All claims expressed in this article are solely those of the authors and do not necessarily represent those of their affiliated organizations, or those of the publisher, the editors and the reviewers. Any product that may be evaluated in this article, or claim that may be made by its manufacturer, is not guaranteed or endorsed by the publisher.

## Supplementary Material

Supplementary Figure 1
**|** Sensitivity analysis for the FT3 serum levels between the survivors and nonsurvivors.

Supplementary Figure 2 Sensitivity analysis for the FT4 serum levels between the survivors and nonsurvivors.

Supplementary Figure 3 Sensitivity analysis for the TSH serum levels between the survivors and nonsurvivors.

Supplementary Figure 4 Sensitivity analysis for the effect of NTIS on the mortality of COVID-19 patients.

Supplementary Figure 5 Sensitivity analysis for the effect of low TSH serum levels on the mortality of COVID-19 patients.

Supplementary Figure 6 Sensitivity analysis for the FT3 serum levels between the patients with severe and nonsevere COVID-19.

Supplementary Figure 7 Sensitivity analysis for the FT4 serum levels between the patients with severe and nonsevere COVID-19.

Supplementary Figure 8 Sensitivity analysis for the TSH serum levels between the patients with severe and nonsevere COVID-19.

Supplementary Figure 9 Sensitivity analysis for the effect of severity on the probability of low FT3 in COVID-19 patients.

Supplementary Figure 10 Sensitivity analysis for the effect of severity on the probability of low TSH in COVID-19 patients.
