# Supplementary figures and images for: The Association Between COVID-19 and Thyroxine Levels: A Meta-Analysis
Source: Front Endocrinol (Lausanne). 2022 Jan 4;12:779692. doi: 10.3389/fendo.2021.779692 (PMC8763686; doi:10.3389/fendo.2021.779692)

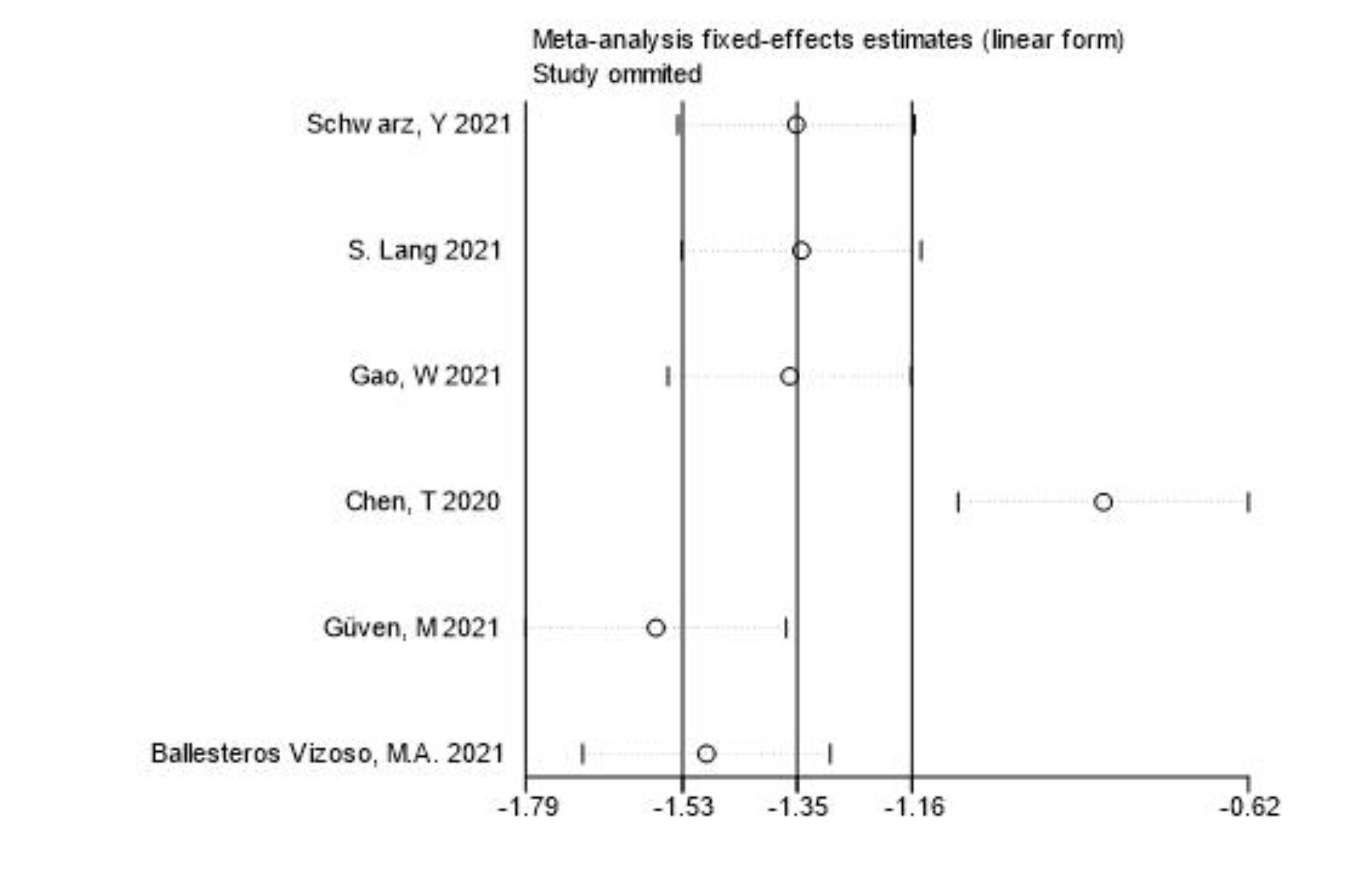

Supplement: Supplementary Figure 1 — | Sensitivity analysis for the FT3 serum levels between the survivors and nonsurvivors. [file Image_1.jpeg]

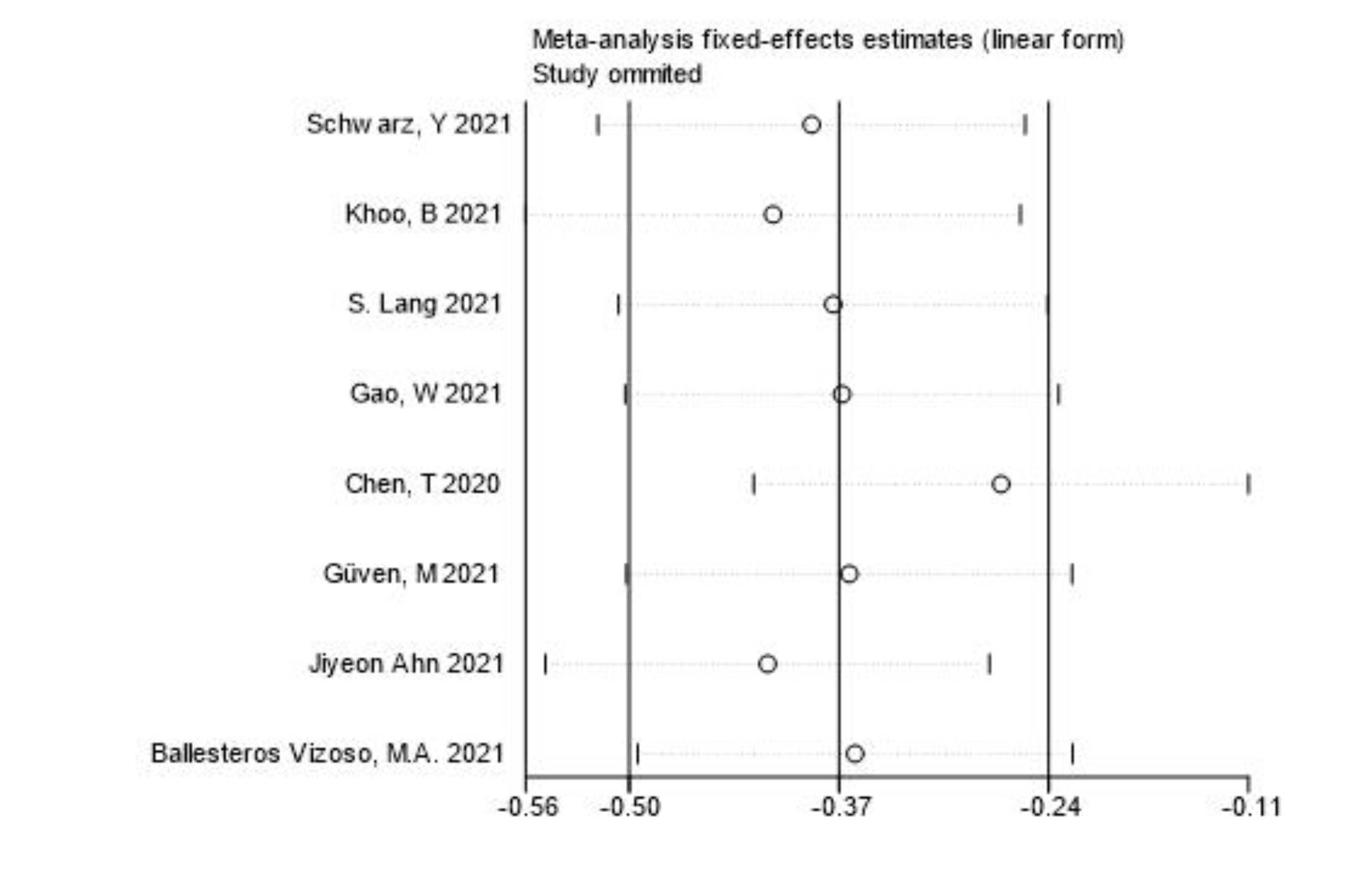

Supplement: Supplementary Figure 2 — Sensitivity analysis for the FT4 serum levels between the survivors and nonsurvivors. [file Image_2.jpeg]

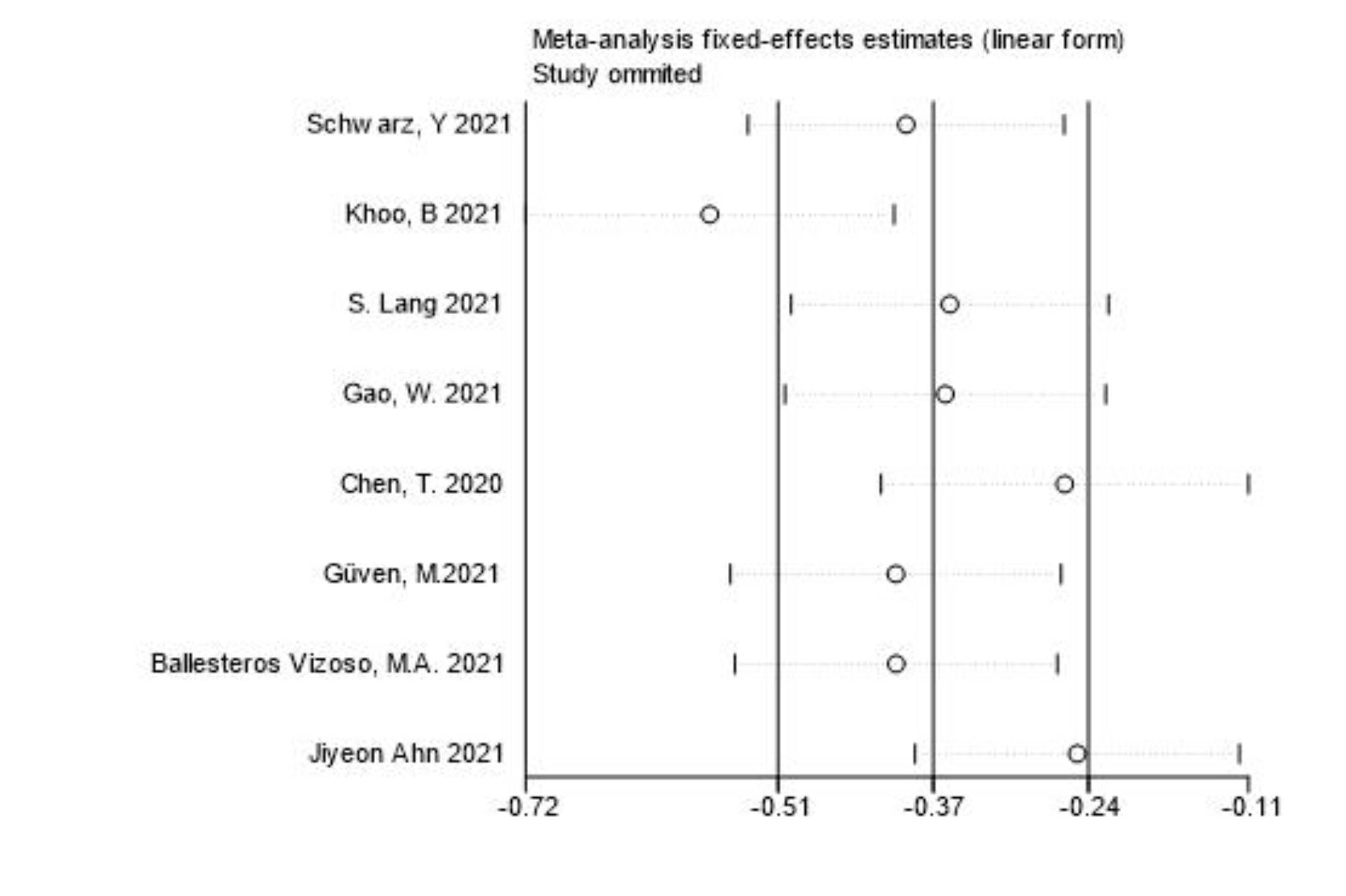

Supplement: Supplementary Figure 3 — Sensitivity analysis for the TSH serum levels between the survivors and nonsurvivors. [file Image_3.jpeg]

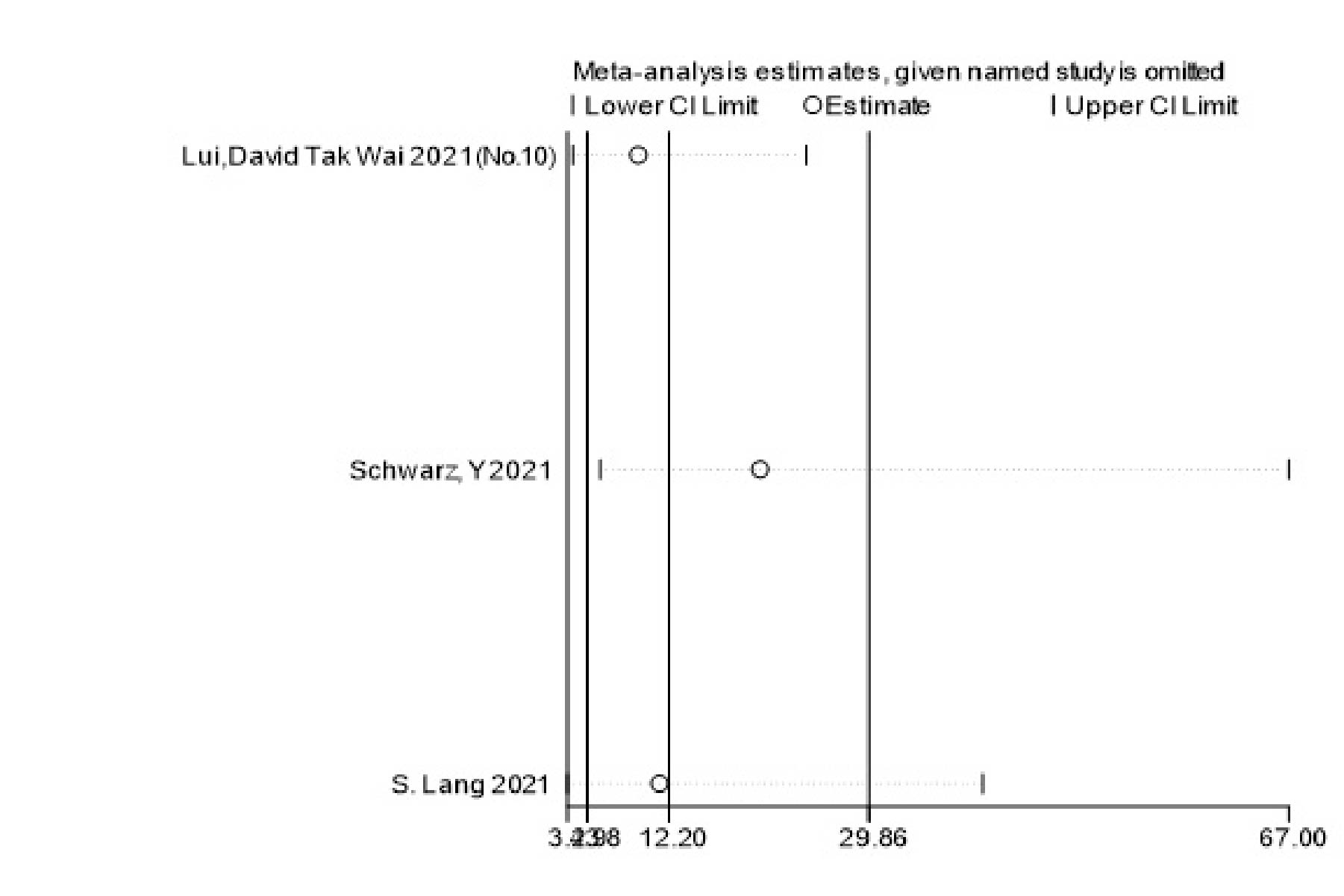

Supplement: Supplementary Figure 4 — Sensitivity analysis for the effect of NTIS on the mortality of COVID-19 patients. [file Image_4.jpeg]

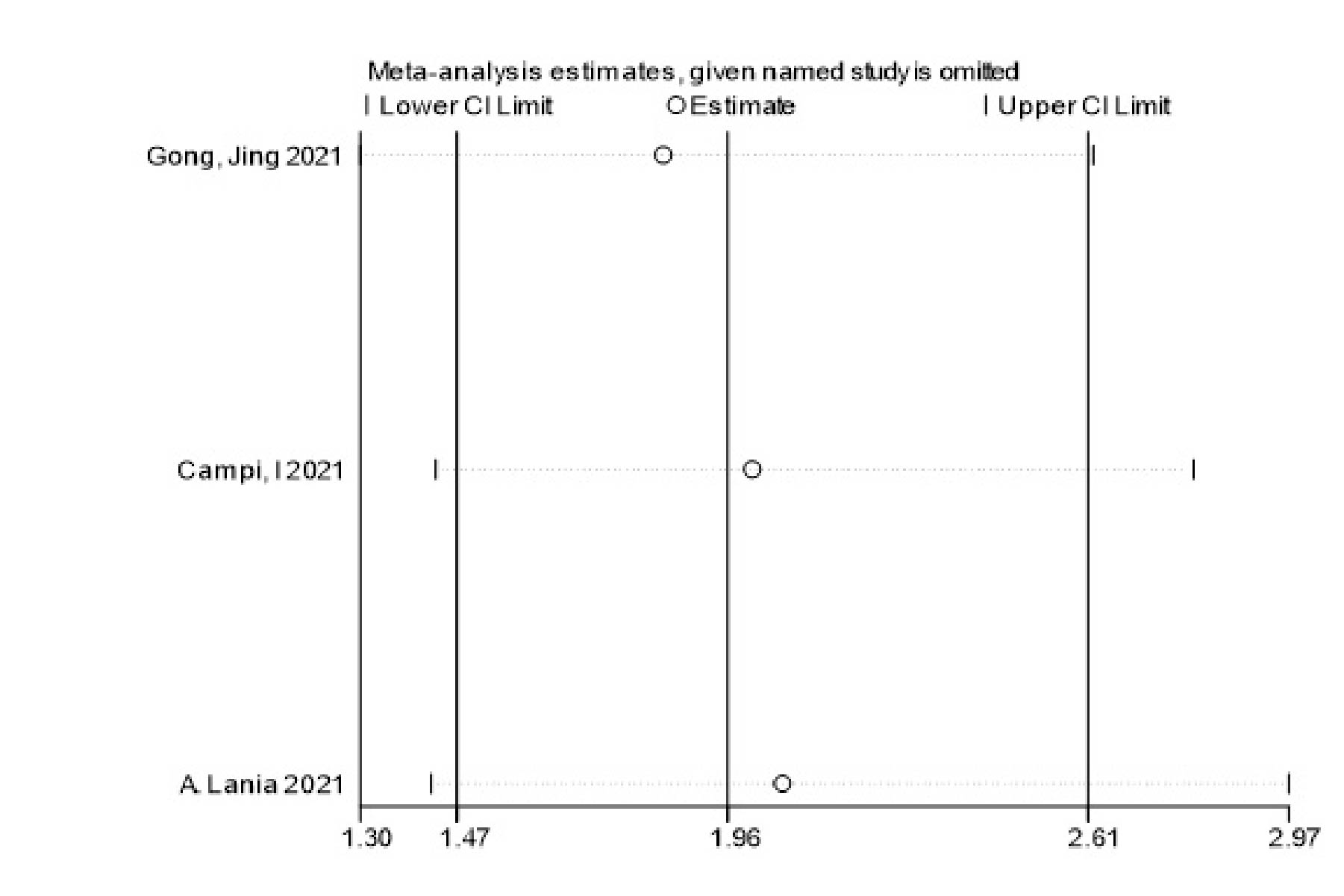

Supplement: Supplementary Figure 5 — Sensitivity analysis for the effect of low TSH serum levels on the mortality of COVID-19 patients. [file Image_5.jpeg]

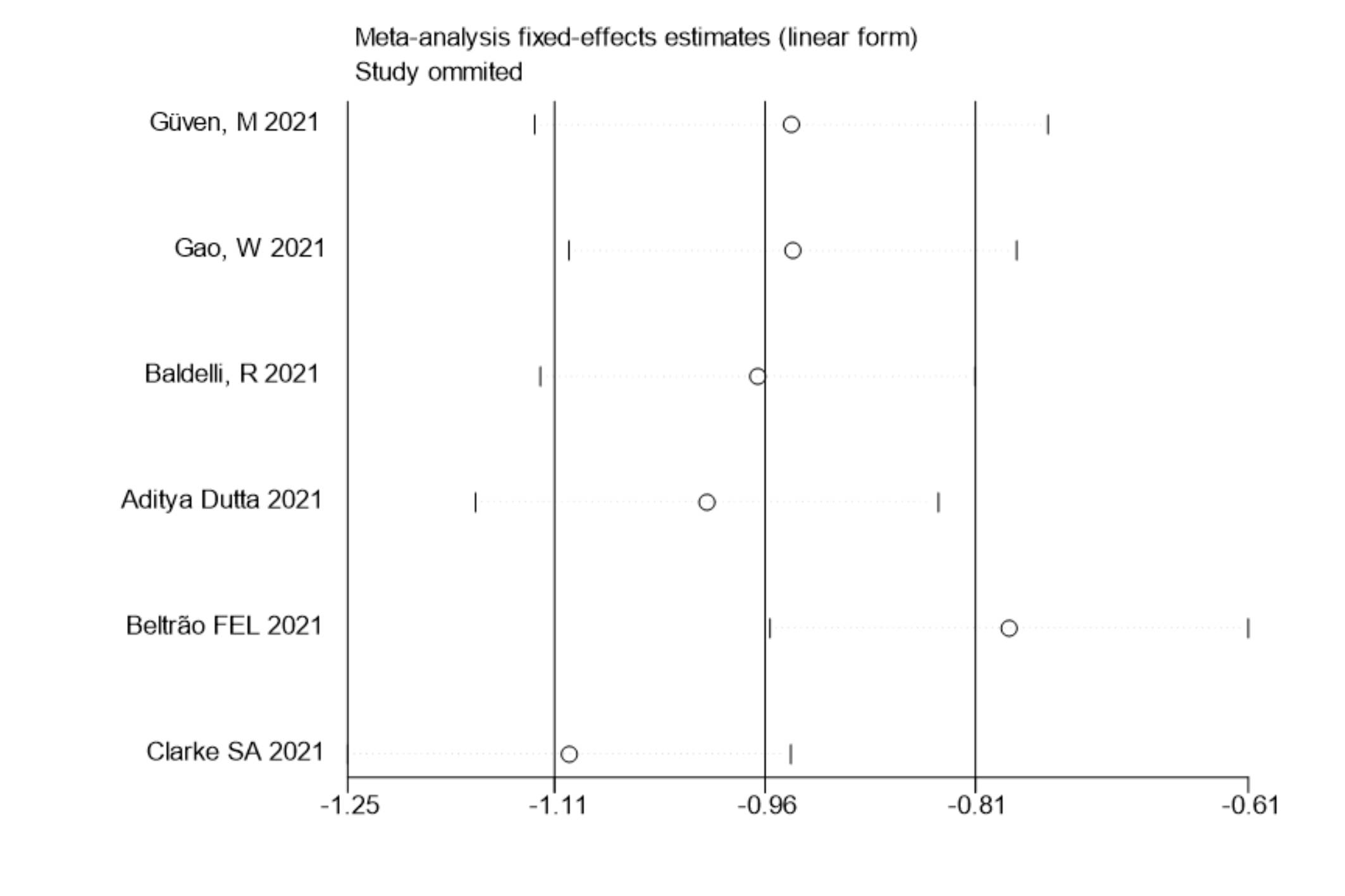

Supplement: Supplementary Figure 6 — Sensitivity analysis for the FT3 serum levels between the patients with severe and nonsevere COVID-19. [file Image_6.jpeg]

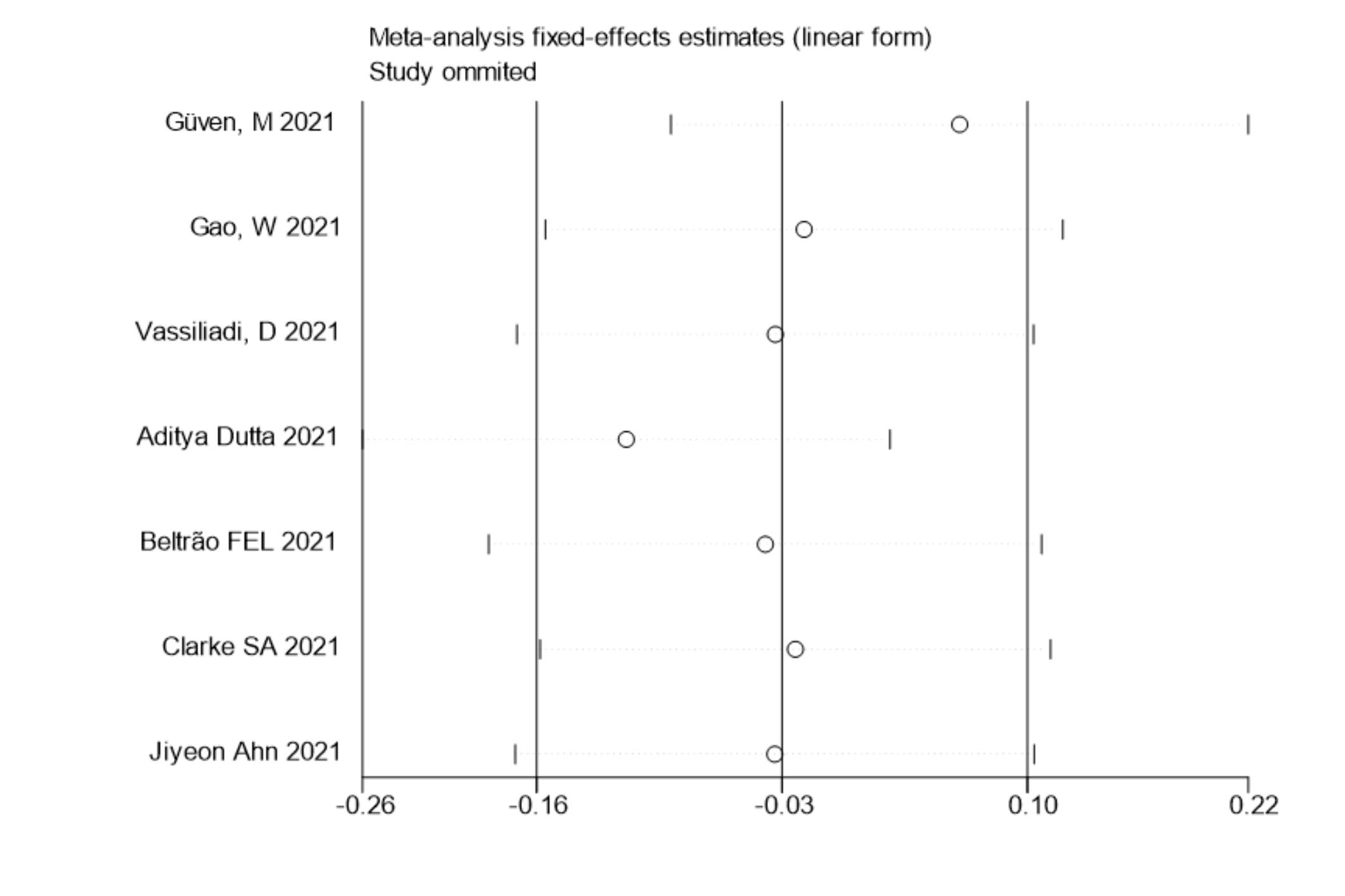

Supplement: Supplementary Figure 7 — Sensitivity analysis for the FT4 serum levels between the patients with severe and nonsevere COVID-19. [file Image_7.jpeg]

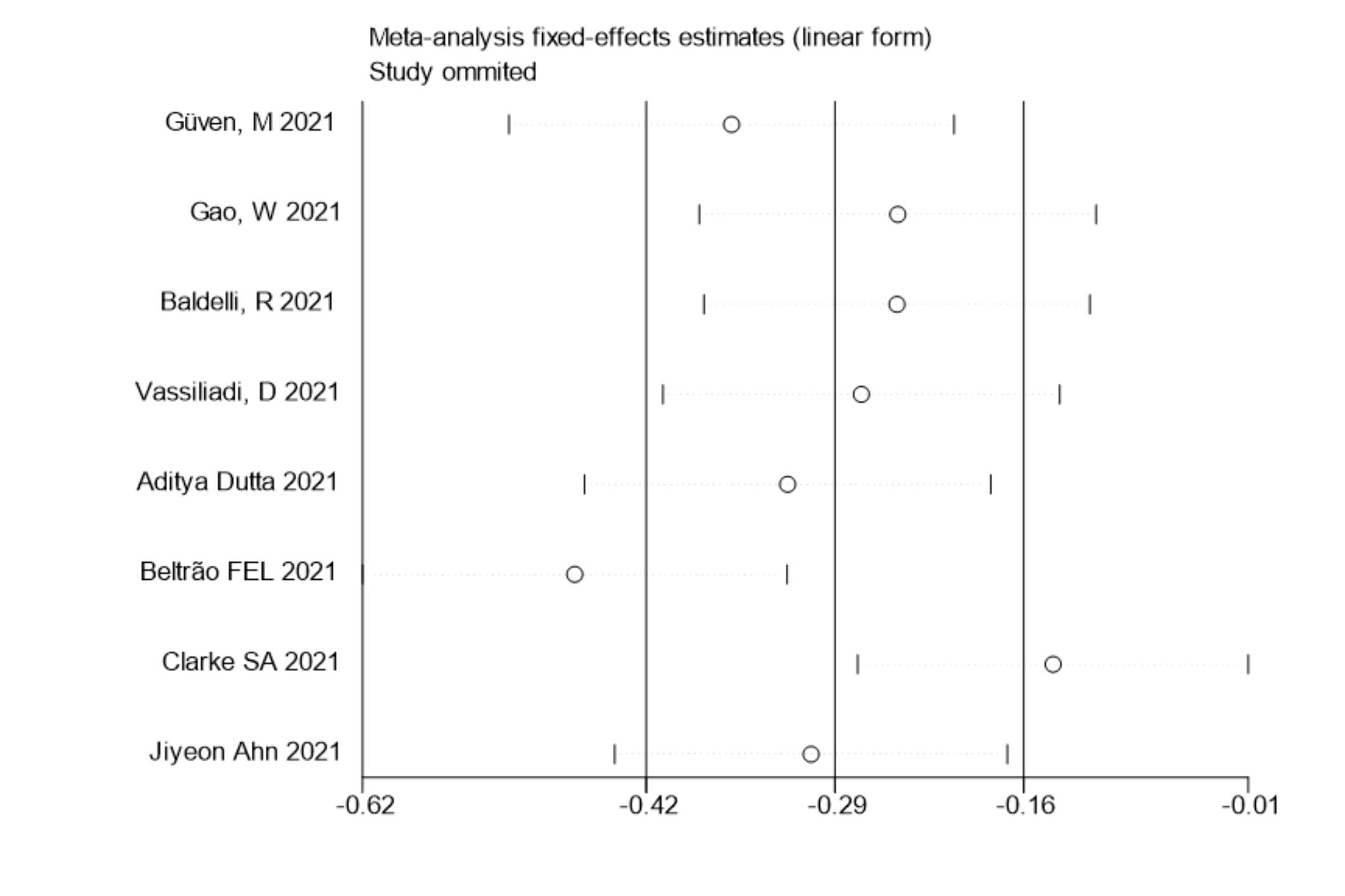

Supplement: Supplementary Figure 8 — Sensitivity analysis for the TSH serum levels between the patients with severe and nonsevere COVID-19. [file Image_8.jpeg]

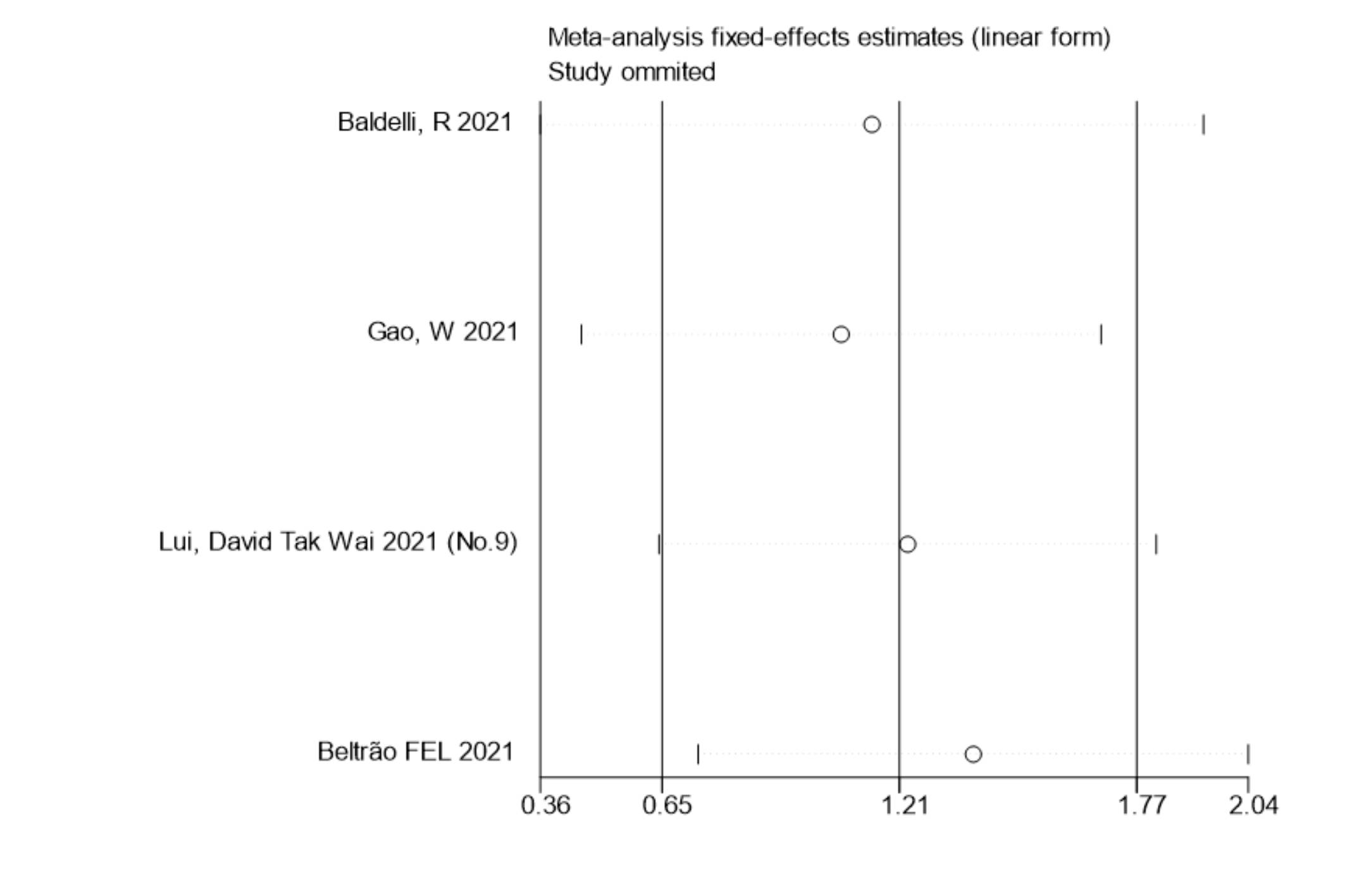

Supplement: Supplementary Figure 9 — Sensitivity analysis for the effect of severity on the probability of low FT3 in COVID-19 patients. [file Image_9.jpeg]

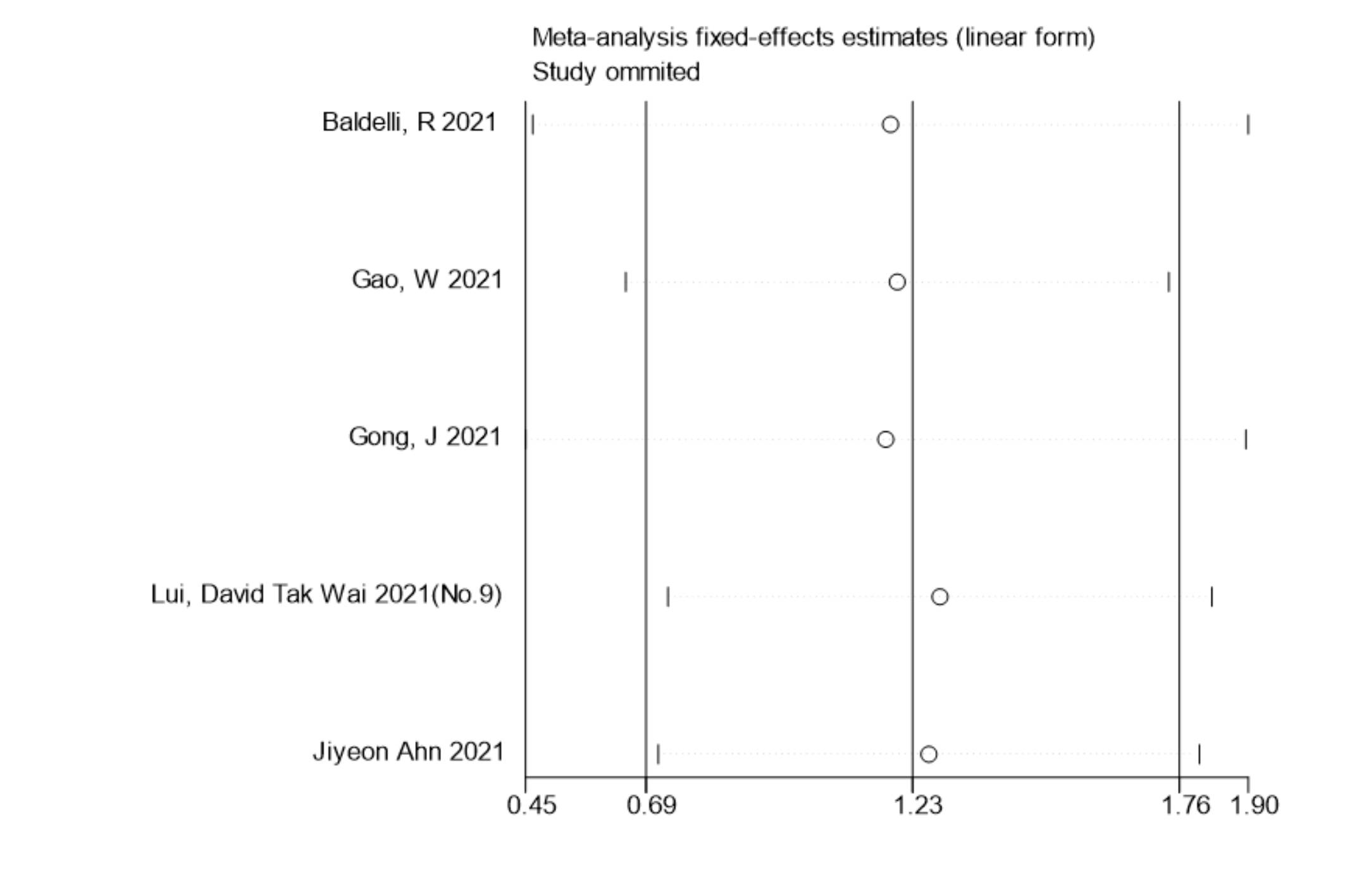

Supplement: Supplementary Figure 10 — Sensitivity analysis for the effect of severity on the probability of low TSH in COVID-19 patients. [file Image_10.jpeg]
